# Empirical retinal venous pulse wave velocity using modified photoplethysmography

**DOI:** 10.1186/s13104-023-06309-y

**Published:** 2023-04-08

**Authors:** Anmar Abdul-Rahman, William Morgan, Aleksandar Vukmirovic, Andrew Mehnert, Danail Obreschow, Dao-Yi Yu

**Affiliations:** 1 Department of Ophthalmology, Counties Manukau DHB, Auckland, New Zealand; 2 Centre for Ophthalmology and Visual Science, The University of Western Australia, Perth, Australia; 3 Lions Eye Institute, University of Western Australia, Perth, Australia; 4 International Centre for Radio Astronomy Research (ICRAR), University of Western Australia, Perth, Australia; 5 International Space Centre, University of Western Australia, Perth, Australia

**Keywords:** Ophthalmodynamometry, Retinal venous pulse, Modified photoplethysmography

## Abstract

**Objective:**

Using the novel imaging method of high-speed modified photoplethysmography we measured the retinal venous pulse wave velocity in a single case.

**Results:**

A healthy 30-year-old subject underwent high-speed modified photoplethysmography (120 frames per second) with simultaneous ophthalmodynamometry at 26 Meditron units. A video of the optic nerve was analyzed using custom software. A harmonic regression model was fitted to each pixel in the time series and used to quantify the retinal vascular pulse wave parameters. Retinal venous pulsation at the optic disc was observed as a complex dynamic wall motion, whereas contraction commenced at a point in the vein at the center of the optic disc, and progressed centrifugally. The empirically estimated retinal venous pulse wave velocity at this segment was approximately 22.24694 mm/s. This measurement provides an estimate for future studies in the field.

## Introduction

### Background

Although the eye provides unique access to the vascular system in vivo several practical limits exist in the measurement and analysis of retinal vascular imaging data. They include the pathway of the retinal vascular system, which traverses two pressurized chambers, the eye and cerebrospinal fluid space, the small dimensions of the vessels, variable vascular geometry, the low signal-to-noise ratio, and imaging artifacts. Additionally, vascular pulse wave velocity (PWV) studies are reduced models because several parameters like pressure, flow, cardiac output, blood pressure, intraocular pressure, and heart rate cannot be measured simultaneously. Several investigators estimated pulse wave velocity from theoretical and experimental models in both human and animal studies. Results range from 0.442 to 620 mm/s in human studies [1, 2], and 98 to 1000 mm/s in animal studies [3–5]. In earlier research, we described a novel imaging method of modified photoplethysmography, which uses a combination of induced intraocular pressures generated by ophthalmodynamometry and slit lamp optic nerve imaging. High-quality video recordings are taken, which are then subject to frame alignment, then pixel-by-pixel analysis over time to objectively extract pulsation metrics using harmonic analysis without requiring an observer and so are not subject to observer bias. Analysis of the pulse signal using this method involves deriving a harmonic regression model [6–12]. The latter emulates the photoplethysmography wave components [13]. The model consists of a linear spline, which adjusts the mean of the signal accounting for inter-frame image displacement. The first two harmonics of the Fourier series are fitted to the periodic component together with an error-correcting first-order autoregressive component. Frequency domain decomposition is performed using custom software, where heat maps of the retinal vascular pulse amplitude distribution are generated. Advantages of this method of other techniques of pulse wave measurement include modeling the retinal arterial and venous systems separately using this method. Uniquely, frequency domain analysis allows computationally efficient information filtering and comparative processing of the retinal vascular pulse characteristics [6, 8, 14]. We hypothesize that signal denoising using frequency domain methods will enable a more realistic benchmark of the retinal vascular pulse velocity. The PWV is traditionally approximated by the wave “foot-to-foot” velocity, which is the distance/time either between sequential wave minimums or between the electrocardiogram R wave/pulse oximetry and the wave minimum [15]. In this study, we empirically estimate the PWV at the optic disc made from the transit time delay of the “onset” or the “foot” between two visible pulse waves measured at two different sites along the pulse propagation path [15]. All parameters, when approximated, are computed to six decimal places.

## Main text

### Materials and methods

Written consent was obtained from the subject. Study approval was obtained from the University of Western Australia Human Ethics Committee, adhering to the tenets of the Declaration of Helsinki. A healthy 30-year-old female subject underwent slit-lamp ophthalmic examination and mPPG of the left eye. The subject was emmetropic. There was no history of systemic or ocular disease. Slit lamp examination did not reveal retinal vascular or optic nerve pathology.

### Ophthalmodynamometry technique

An imaging slit-lamp (Carl Zeiss, Germany) with a digital camera (Canon 5D Mark III, Japan) was used to record multiple video sequences of at least three cardiac cycles in length, each at a rate of 120 FPS frames/second. Simultaneous Meditron ophthalmodynamometry (Meditron GmbH, Poststrasse, Völklingen, Germany) was undertaken. The Meditron ophthalmodynamometer consists of a three-mirror Goldmann contact lens fitted at the observer end with a ring-shaped force transducer. The sensor ring is connected to a liquid crystal display monitor, on which the force continuously measured by the sensor ring is displayed. A foot pedal is connected to the display monitor, which communicates the ophthalmodynamometric force to the display. The examination commenced with the calibration step, the observer end of the Goldman contact lens was rested on a flat surface and the device was activated. After initialization, an audio signal indicated a successful calibration. Baseline intraocular pressure was measured, and the subjects’ pupils were dilated with 1% Tropicamide. The Goldmann contact lens of the ophthalmodynamometer was placed on the topically anesthetized corneal surface. Contact gel was applied to the lens to ensure optical interface continuity with the cornea. The examination was performed by applying gradual increments in pressure onto the contact lens up to a constant force at the observers’ 26 Meditron units of ophthalmodynamometric force. The aim was to induce visible pulsations in the central retinal vein and maintain a constant force. The force measured by the sensor ring surrounding the Goldmann contact lens was then read by pressing on the foot pedal; a second observer noted the displayed reading. The optic nerve head was continuously imaged bio-microscopically during the examination through a Meditron Ophthalmodynamometer. A pulse oximeter (Nellcor N65, Covidien, Mansfield, MA) was applied to the right index finger; the audio signal from the pulse oximeter, captured with the video sequence of the optic nerve, allowed synchronization of the retinal vascular pulse with the cardiac cycle. The video, in this case, was recorded at an ophthalmodynamometric force of 26 Meditron units for 10 s, from which a spliced video segment of 2.56 s was analyzed to span three consecutive cardiac cycles. Timing attributes captured by the custom software include the cardiac cycle time and time to the minimum point of the harmonic regression wave (time to trough) measured in fractions of the cardiac cycle. Individual image frames were extracted from each video sequence and saved as Tagged Image File Format (TIFF) files using Adobe Photoshop CS6. Each of these images was cropped to an array of pixels [8].

### Mathematical model

Image analysis and segmentation are detailed in our previous work [6, 8]. A harmonic regression model was fitted to each pixel in the time series and used to quantify the retinal vascular pulse wave parameters including the harmonic regression wave amplitude (HRW$_{\text {a}}$). The model includes a Fourier series representation using the first and second harmonics, a linear spline non-periodic component, and a first-order autoregressive error component. In our model each data point represented by the mean of the green channel intensity (y(t)) at time (t) is measured as a fraction of the cardiac cycle. Equation 1 represents the nominal time for frame (i) in cycle (c, where c=1–3).

1 $$\begin{aligned} { t }_{ i }=\frac{ i }{ { n }_{ c } } +c-1 \end{aligned}$$

where n$_{\text {c}}$ = number of frames in the cth cycle.

The components of the series were expressed as a sum of the trend or regular term (f(t)) and the stationary error, irregular or residual term ($\epsilon _{\text {t}}$) with a zero mean (Equation 2).

2 $$\begin{aligned} y\left( t \right) =f\left( t \right) +{ \epsilon }_{ t } \end{aligned}$$

The trend component was decomposed into periodic f(t)$_{\text {p}}$ and non-periodic components f(t)$_{\text {np}}$, the latter is to account for changes in intensity due to subject movement artifact (Equation 3).

3 $$\begin{aligned} f\left( t \right) ={ f\left( t \right) }_{ p }+{ f\left( t \right) }_{ np } \end{aligned}$$

The periodic trend component was modeled as a Fourier series expansion (Equation 4), this approach is used in the field of computational fluid dynamics of oscillating flow [16, 17].

4 $$\begin{aligned} {{\mathscr {F}}}({ f\left( t \right) }_{ p }) ={ a }_{ 0 } +\sum _{ n=1 }^{ \infty }{ { a }_{ n }\cdot cos({ n\pi t }) + { b }_{ n }\cdot sin({ n\pi t })} \end{aligned}$$

- a$_{\text {0}}$ = Coefficient representing the mean of f(t)$_{\text {p}}$.
- a$_{\text {n}}$ = Coefficient of the cosine function of f(t)$_{\text {p}}$.
- b$_{\text {n}}$ = Coefficient of the sine function of f(t)$_{\text {p}}$.
- n = Integer 0,1,2... etc. representing the harmonic component.

Higher harmonic frequency model comparisons were conducted using the Akaike Information Criterion (AIC). In most eyes AIC preferred models with first and second-order frequencies (Equation 4). Therefore the final analysis was limited to the first and second harmonics [14].

The non-periodic component of the trend f(t)$_{\text {np}}$ was modeled using a linear spline with knots at times t=1 and t=2 (equation 5). Knot frequency was based on the observation that the duration of most artifactual movements is at least one second, which in turn is approximately equal to one cardiac cycle.

5 $$\begin{aligned} { f\left( t \right) }_{ np }={ b }_{ 0 }+{ b }_{ 1 }t+{ b }_{ 2 }{ \left( t-1 \right) }_{ + }+{ b }_{ 3 }{ \left( t-2 \right) }_{ + } \end{aligned}$$

where the subscript + indicates truncation below at zero, so that z$_{{+}}$=z for z>0 and z$_{{+}}$=0 for z$\le$0 [14]. The error component of the series described by equation 1 was modeled using a first- order autoregressive process (i.e. the value of the point in the series is weighted by a value of a proceeding datapoint, separated by one lag in the time series), so as to account for residual serial dependence in the data.

6 $$\begin{aligned} \epsilon _{t}=\rho \epsilon _{\text {t-1}} + u_{\text {t}} \end{aligned}$$

where u$_{\text {t}}$ is white noise and $\rho$ is the autoregressive parameter estimated by restricted maximum likelihood (REML).

### Results

**Fig. 1:**
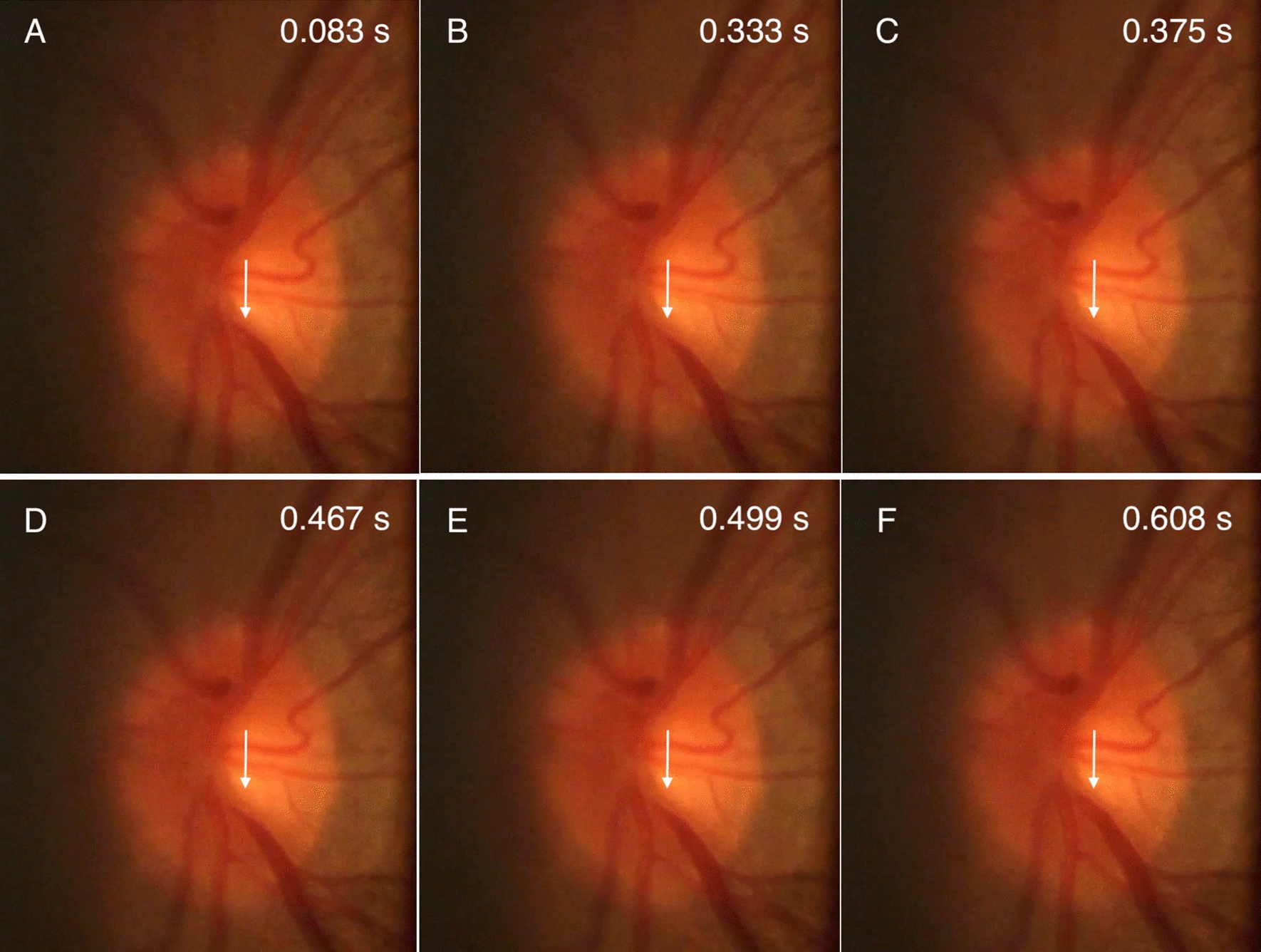
Selected optic disc frame sequence captured during modified ophthalmodynamometry. The inferior retinal vein, initial contraction** A**–** C** and dilation of the segment** D**–**F**. Time was measured from pulse oximetry audio signal. At 120 frames per second (s) each frame represents approximately 0.008333 s. The total contraction time of the inferior retinal vein was estimated at 0.424983 s

The sequence of retinal images (Fig. 1) demonstrated contraction of the proximal retinal vein centrifugally. As the frame rate is 120 FPS, therefore time representation for each frame = 1/FPS=1/120 $\approx$ 0.008333 s (s). Pulsations in the lower venous segment occurred between frames 221 and 272 corresponding to 51 frames. Therefore the duration of a full visible pulsation cycle of the inferior temporal vein is 51*0.008333 $\approx$ 0.424983 s.

**Fig. 2:**
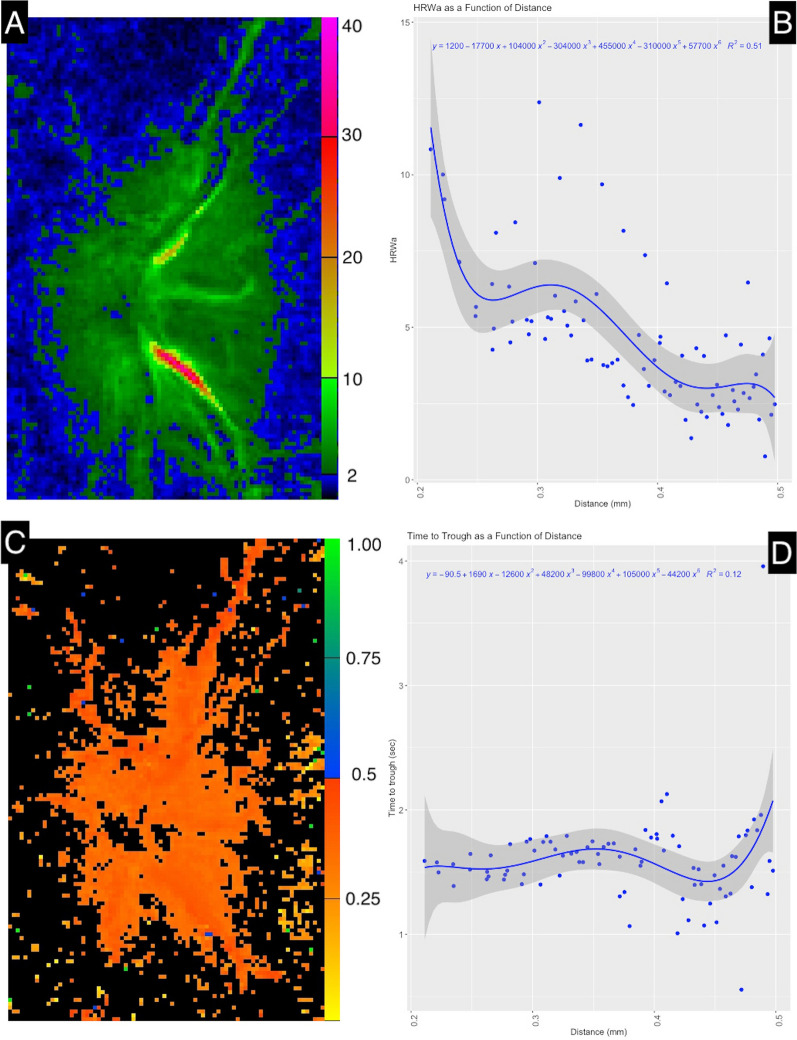
**A** Harmonic regression wave amplitude (HRW$_{\text {a}}$) for the whole image field.** B** HRW$_{\text {a}}$ along the imaged inferior venous segment up to 0.5 mm from the center of the optic disc.** C** Time to harmonic regression wave minimum for the whole image field in fractions of the cardiac cycle.** D** Time to harmonic regression wave minimum along the imaged inferior venous segment up to 0.5 mm from the center of the optic disc

**Fig. 3:**
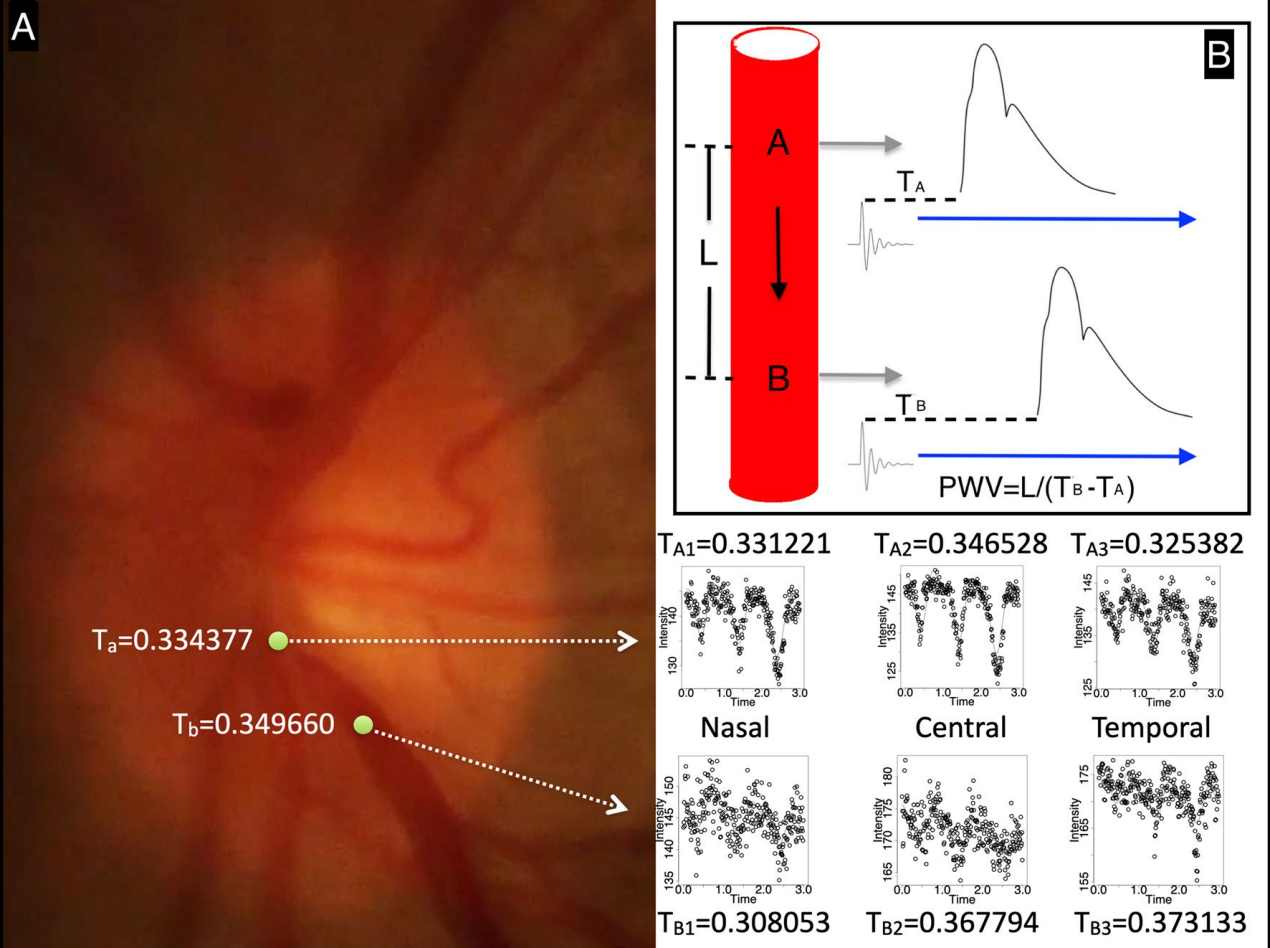
**A** Time to the trough of the pulse wave originally measured as a fraction of the cardiac cycle and converted to seconds by multiplication by the cardiac cycle time. The harmonic regression wave fit of three consecutive cardiac cycles is shown at 3 horizontal points along the venous wall nasal, central, and temporal at two locations proximal (**A**) and distal (**B**) to the optic disc spanning the visible pulsatile segment distance spatially calibrated to a vertical disc diameter of 1.65 mm was 0.34 mm. Pulse transit time was calculated by subtracting the time to trough of the harmonic regression wave at the proximal (T$_{\text {a}}$=0.334377) and distal point (T$_{\text {b}}$=0.349660) along the vessel, each computed from the average of T$_{\text {A1-3}}$ and T$_{\text {B1-3}}$ respectively, located on the nasal, central and temporal vein wall. Pulse Transit Time = 0.349660 - 0.334377 = 0.015283 s. Pulse wave velocity = Distance / Pulse Transit Time = 0.34/0.015283 $\approx$ 22.24694 mm/s.** B** Schematic of the time to trough measured as the distance between the pulse oximetry audio and the foot of the pulse wave at two points along the path of pulse wave propagation

Individual frames were spatially calibrated in ImageJ [18], and the distance along the vessel was scaled based on an assumed optic disc measurement of 1.65 mm. The geodesic estimated distance was 0.34 mm. Figure 2 demonstrates the distribution of HRW$_{\text {a}}$ and time to trough (harmonic regression wave minimum) throughout the entire image field and along the inferior retinal vein. Two points were selected on the inferior retinal vein on either side of the contractile segment, (A) proximal and (B) distal to the optic disc as shown in Fig. 3. Time to trough at two points proximal to the optic disc (T$_{\text {a}}$) and distal to the optic disc (T$_{\text {b}}$) was calculated as the time in fractions of the cardiac cycle between the pulse oximetry audio signal and the foot of the harmonic regression wave at three points for each of T$_{\text {a}}$ and T$_{\text {b}}$, located on the nasal, central, and temporal venous wall (T$_{\text {A1-3}}$, T$_{\text {B1-3}}$). For each of the three points time to trough was converted to seconds by multiplying by the cardiac cycle time (0.852743 s) and calculating the average of T$_{\text {A1-3}}$ and T$_{\text {B1-3}}$, yielding T$_{\text {a}}$ =0.334377 s and T$_{\text {b}}$=0.349660 s respectively. Pulse transit time was calculated by subtracting the time to trough at the distal (T$_{\text {b}}$) and proximal points (T$_{\text {a}}$) along the vessel. Therefore the pulse transit time = 0.349660 - 0.334377 = 0.015283 s. PWV = Distance / Pulse Transit Time = 0.34/0.015283 $\approx$ 22.24694 mm/s.

## Discussion

To our knowledge, no studies have reported human retinal venous PWV. We found the observable inferior retinal venous PWV $\approx$ 22.24694 mm/s. Our estimate was constrained by the temporal limit (the duration of each video frame), a time scale constraint is imposed at 0.008333 s, and the spatial constraint was due to the maximum segment length of the lower retinal vein in the image frames (1.197 mm). Therefore, a maximum velocity of 1.197/0.008333 = 143.646 mm/s can be estimated in this image field. A literature review is summarized in Table 1, wherein appropriate scrutiny should be exercised due to methodological differences. In the systemic circulation, the PWV in the microvessels is observed to be two orders of magnitude slower than in the aorta [19]. These experimental findings are consistent with linear pulse wave transmission theory in a branching system of vessels. Salotto et al. [20] reported that the PWV alters from a value in the order of meters/second in large arteries to a value in the order of centimeters/second in the microvessels. Otto reported quantitative data on PWV in retinal arteries by analysis of motion-compensated angiography image sequences [21]. He reported retinal arterial PWV in healthy subjects ranging between 24 mm/s in a 30-year-old, 240 mm/s in a 63-year-old, and >300 mm/s in a 45-year-old patient with arterial hypertension.

**Table 1:** Studies of retinal pulse wave velocity. Animal studies were on rodents with vessels < 300 microns in diameter

	Year	Method	Velocity (mm/s)
Human studies			
Otto [21]	1999	FFA	24-174
Kotliar et al. [22]	2011	DVA	21-243
Kotliar et al .[1]	2013	DVA	0.442
Spahr et al. [2]	2015	OCT	620
Li et al. [25]	2018	OCT	20-30
Rezaeian et al. [24]	2020	DVA	25
Animal studies			
Seki [27]	1994	Fiber optic anemometer microscope	452
Yeh et al. [5]	2012	Photoacoustic microscopy	1000
Golzan et al. [3]	2014	Optronic camera	98-114
Song et al. [4]	2016	OCT	1000

All listed studies are arteriolar pulse wave velocity.

*FFA* Fluorescein angiography,* DVA* Dynamic vessel analyzer,* OCT* Optical coherence tomography

Using the dynamic vessel analyzer (DVA), Kotliar et al. estimated the retinal PWV in 20 subjects classified by age into young (median age 26 years) and senior (median age 67 years). A 60-s recording of retinal arterial diameter changes was captured at a distance of one and three-disc diameter regions. PWV was calculated as the ratio between the distance of analyzed segments divided by the phase shift between points of interest. Estimated PWV ranged between 21.5 (17.9$-$4.6) mm/s in young subjects, and 243.8 (186.1$-$347.7) mm/s in elderly [22]. This suggested a large age dependence. They subsequently revised the value in a follow-up study to 0.410 (0.280$-$0.500 mm/s) in young subjects and 0.400 (0.320$-$0.510) mm/s in healthy seniors [23]. Rezaeian et al. compared the carotid-femoral PWV (cfPWV) and PWV. The slope of the regression between phase difference (radians) and angular frequency (radians/s) measured at the two points was considered the pulse transit time (PTT), and this was measured at a distance of $610\mu \hbox {m}$ along the vessel. Systolic blood pressure, Diastolic Blood Pressure (DBP), carotid-femoral PWV (cfPWV), and IOP (transmural pressure =DBP - IOP) were measured to assess correlation with PWV using multivariate lasso regression. PWV was estimated at 25±15 mm/s. Only cfPWV and IOP achieved statistical significance in correlation with PWV [24]. Limitations in the image resolution of DVA and a corresponding sampling rate of 25 frames/second restrict measurement accuracy as each frame represents 1/25 = 0.04 s, representing the minimum time scale. The spatial window also limits PWV, whereas a measurement separation between vascular points of 4.5$-$9.5 mm limits the PWV to 4.5/0.04=112.5 mm/s to 9.5/0.04 = 237.5 mm/s [22], a spatial window of 610$\mu$ limits the maximum velocity to 610/(1000*0.04) = 15.25 mm/s

Optical coherence tomography was used in two studies; Spahr et al. [2] used phase-sensitive full-field swept-source optical coherence tomography of areas of interest on the vessels separated by 11-12 mm. The local retinal pigment epithelial and retinal nerve fiber layer expansion rate was calculated by measuring the phase shift between pulse waves. Foot-to-foot distance of the retinal arteriolar pulse was measured by estimating the time delay based on the phase shift of the pulsation curves band-pass filtered at the cardiac frequency. The authors reported a retinal arterial PWV = 620±50 mm/s,. However, the venous pulse could not be determined using this method as a time delay between the pulsations of the two venous segments could not be detected. Li et al. [25] used spectral-domain optical coherence tomography and used the foot-to-foot time as the pulse period. The elongation of a cardiac pulse period observed in a Doppler-OCT jump-scan measurement was evaluated to estimate the time required by the pulse wave to propagate the distance of the jump. Using this method, they observed pulse wave velocities of 20 to 30 mm/s in a healthy subject. The PWV observed in a prehypertensive subject was approximately 50 mm/s. As the jump-scan method compares the length of sequential cardiac cycles, the accuracy of this method is therefore limited by the natural variations of the length of the cardiac cycle; the lengths of directly adjacent cardiac cycles are known to vary by typically 35±20 ms [26]

The propagation of pulse waves in vessels with a diameter below 300 $\mu$m was investigated in animal studies using various techniques. Seki [27] compared the PWV in microvessels of rats using a fiber-optic laser Doppler anemometer microscope. Whereas arteriolar flow propagation velocity ranged between 3.5 - 134 cm/s in arterioles of 12-43 $\mu$m in diameter, increasing linearly with increasing diameter, in venules, the range was 4.8–16.1 cm/s for vessels 35–55 $\mu$m in diameter. Wang et al. used a photoacoustic approach in mice. They reported an average propagation velocity of 1000 mm/s in vessels with a diameter between 50 and 80 $\mu$m [5]. In 2014 Golzan et al. [3] determined the PWV in the retina of rats using high-speed video imaging with simultaneous ECG. They measured an average propagation velocity of 1140±61 mm/s. Song et al. [4] observed pulse waves propagating at more than 1000 mm/s in the retina of rats using high-speed swept-source OCT.

It can be noted that human studies report arteriolar PWV values that vary by a factor of 1550. When animal studies are considered, this variation increases to a factor of 2500. This suggests that the velocity is either highly sensitive to the experimental methodology or reduced PWV models may reflect wide variations in other unmeasured parameters. Therefore reproducible approaches with a high signal-to-noise ratio are required that can be supported by theoretical and physical models. To achieve this goal, limited by practicality, computations require concomitant measurements of local factors, including intraocular pressure, properties of the vascular wall pulse, pressure, and flow waves, as well as an estimate of both the retinal vascular incremental modulus of elasticity and vessel wall density. Additionally, systemic factors, especially cerebrospinal pressure wave spatial and temporal properties, heart rate, and blood pressure, need to be considered.

Pulse wave velocity is emerging as the gold standard for evaluating vascular compliance as an independent predictor of coronary heart disease, stroke, and mortality, [28–31] Several studies have applied retinal vascular characteristics as an indirect biomarker for systemic vascular compliance. Observational studies linked cardiovascular mortality [32], hypertension [33], coronary heart disease [34], and lacunar stroke [35] to retinal vascular parameters have mainly concentrated in three approaches:

1. Retinal vascular caliber using conventional fundus photography [36–41].
2. Retinal arteriolar maximum-to-minimum velocity ratio or wall-to-lumen ratio using scanning laser Doppler flowmetry and automatic full-field perfusion imaging [42, 43].
3. Retinal vascular fractal dimensions [44, 45].

However, these approaches fail to study the primary site of the pathological change, which is the vessel wall, in isolation. The retinal vascular pulse represents the final common pathway in interactions between the hemodynamic pressure-flow wave, vessel wall tension, and compliance [6]. We demonstrated that modified photoplethysmography can potentially provide an avenue to address this gap.

### Conclusion

We measured retinal venous PWV from an observable pulsatile peripapillary segment. This measurement provides an estimate for future studies in the field.

## Limitations

The analysis is from a pulsatile retinal venous segment in a single case. Future approaches will involve deriving a generalized model.

## Data Availability

All relevant data are within the manuscript. Further inquiries should be addressed to Dr Anmar Abdul-Rahman (anmar_rahman@hotmail.com).
